# Supplementary figures and images for: Coronavirus M protein disperses the trans-Golgi network and inhibits anterograde protein trafficking in the secretory pathway
Source: PLoS Pathog. 2026 May 5;22(5):e1014117. doi: 10.1371/journal.ppat.1014117 (PMC13167032; doi:10.1371/journal.ppat.1014117)

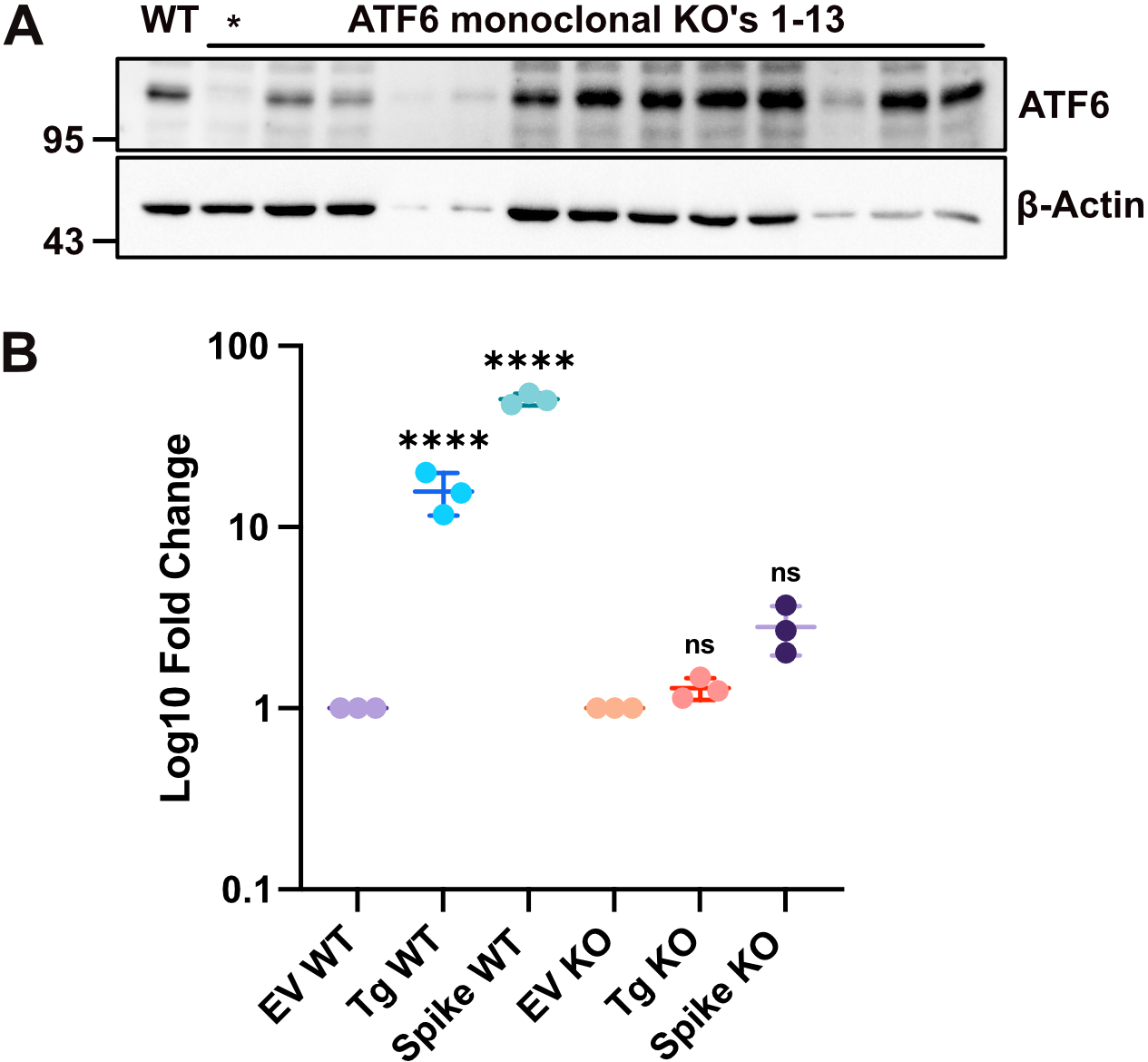

Supplement: S1 Fig — (A) HEK293T cells were transduced with lentiviruses encoding pLentiCRIPSRv2-ATF6 constructs to knockout the ATF6 gene. At 24 h post-transduction cells were selected in 10 mg/mL of puromycin. Cells were seeded in a 96-well plate to generate monoclonal populations and expanded under puromycin selection. Selected monoclonal populations were seeded into 6-well plates, lysates were harvested 48 hours post-seeding and stored at -20C prior to immunoblotting. * indicates the knockout clonal population selected for further experimental use. (B) Wild-type HEK293T or ATF6-KO cells were transfected with Spike or treated with Tg for 1h prior to harvest. (n = 3 ± SD, statistical significance was determined by one-way ANOVA with Fisher’s LSD test. ****, adj. P < 0.00001 relative to EV WT or EV KO.). (TIFF) [file ppat.1014117.s001.tiff]

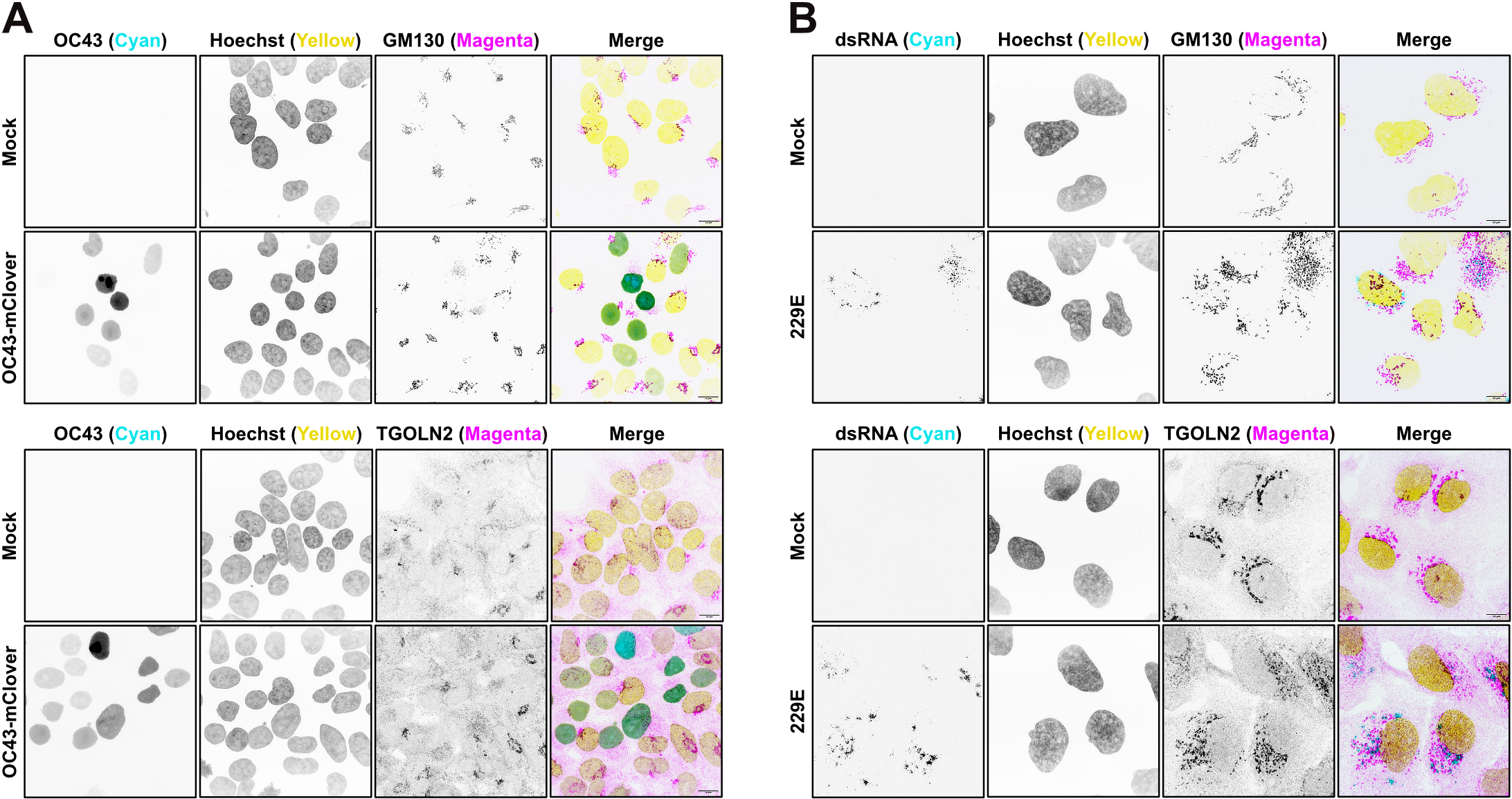

Supplement: S2 Fig — Confocal immunofluorescence images of GM130 (cis-Golgi), or TGOLN2 (trans-Golgi) in coronavirus infected cells. (A) HEK293T cells were infected with HCoV-OC43-mClover at an MOI of 0.05 then fixed at 24 h post-infection. Infected cells were infected with HCoV-229E at an MOI of 0.05 then fixed at 24 h post-infection. Infected cells were identified with the J2 monoclonal antibody that binds dsRNA. Maximum intensity projections are presented. 100X magnification, scale bar = 10 µm. Representative images of three independent experiments. (TIFF) [file ppat.1014117.s002.tiff]
