# Supplementary material for: Coronavirus M protein disperses the trans-Golgi network and inhibits anterograde protein trafficking in the secretory pathway
Source: PLoS Pathog. 2026 May 5;22(5):e1014117. doi: 10.1371/journal.ppat.1014117 (PMC13167032; doi:10.1371/journal.ppat.1014117)
Supplement: Attachment — Submitted filename: Response to Reviewers - CoV M - Feb 27 2026.docx [file ppat.1014117.s004.docx]

**Summary of improvements:**

1. In response to critiques from both Reviewer 2 and Reviewer 3, we extensively revised Fig. 6, which now features two cholesterol probes, D4H-mCardinal, and the new BODIPY 480/508-cholesterol (BCh2), that both show striking co-localization with SARS-CoV-2 M protein in the *cis*-Golgi. We conducted 3 biological replicates of each of these experiments and used a Pearson’s Correlation Co-efficient test to quantify the strong correlation between M and the cholesterol probes.
2. We have also revised Figure 5 (cargo sorting assay) to provide quantitation of co-localization of M and the mApple-TNF-RUSH cargo reporter protein in the cis-Golgi, which only occurs following biotin addition. The Pearson’s Correlation Co-efficient test strongly supports this conclusion.
3. We have revised the manuscript extensively for greater clarity throughout, modifying text and addressing minor critiques from all three reviewers. We re-wrote the Abstract, and the old Abstract (which was quite brief) is now the Author Summary, which fits better with the style of the journal.

**Editor Comments:**
Consider using singular "Coronavirus M protein" in the title and elsewhere. It applies to all coronaviruses and avoids "M proteins" that could be confusing.

Fixed.

We have changed this description from plural to singular in the manuscript title and in other instances.

Please adhere to the acronym usage as agreed in coronavirus field: NSP -> nsp, hCoV -> HCOV

Fixed.

We have changed acronym formatting as suggested throughout the manuscript.

**Reviewer #1:** This merit of this well-written submission is with its demonstration that coronavirus membrane (M) proteins impede host protein anterograde transport in the exocytic pathway. The findings bring greater insights into published findings showing that coronavirus infections corrupt host exocytic organelles. The demonstration that M proteins operate globally in halting protein cargo transit at the cis Golgi is a significant contribution. The additional novelty – as communicated by authors – is that M proteins can now be viewed as more than the fundamental components of the virion. They can now be viewed as participants in host cell remodeling processes that both limit antiviral responses and foster development of proviral virion budding sites.

This submission begins by testing UPR activation by SARS2 viral proteins. The results showed that three arms of the UPR pathway were activated by spikes (S) [and by chemically-induced protein misfolding] but only the ATF6 arm was reduced by co-expressed M. ATF6 activation requires ATF6 transit to Golgi – hence the authors developed the hypothesis that M blocks ER to Golgi transport. [Note that this hypothesis diverges from the UPR-centric beginning of the paper – however the story is clearly developed by the authors.] The hypothesis was then tested in several ways; M reduced SREBP and STING activation (both operate from Golgi), M reduced Gluc secretion, M accumulated in ERGIC along with cholesterol, RUSH assays showed a reporter protein transport from ER to ERGIC, and no further, when M proteins were present, and M dispersed the later TGN organelles. The results generally supported the conclusion that M accumulation in ERGIC halts protein transport at the ERGIC. This appears to be a worthy report that will be appreciated by cell biologists and virologists.

We are very pleased that Reviewer 1 appreciates the quality our manuscript.

Major Concerns:

1. The vast majority of experiments involved plasmid overexpression in HEK293 cells. While the results were convincing, they may not reflect events taking place in natural infection contexts. For example, suppression of UPR, SREBP2, STING, could come from nsp1 or other viral proteins, moreso than M. Perhaps the discussion section should include communication of the caveats associated with the results coming from reductionist assays in HEK293 cells.

We acknowledge that, as we describe a new function for M protein, there are many other viral proteins that modify host antiviral responses, and even other viral proteins that interfere with the secretory pathway. As the Reviewer suggests, we have modified the Discussion to make this clearer, as follows (lines 308-323):

“*Using a RUSH cargo sorting assay, we observed that M accumulated in the cis-Golgi and inhibited further anterograde transport of a transmembrane reporter protein beyond this compartment, while dispersing the TGN, a phenotype that we also observed during infection with diverse COVs. Together, these observations suggest that COV M protein disrupts the TGN and impedes normal anterograde traffic in the canonical secretory pathway. Because COV egress does not require the TGN, this mechanism could allow the virus to selectively interfere with host responses to infection without impeding egress of nascent virions. This research adds yet another mechanism of host subversion to the impressive COV armamentarium.*

*Golgi dispersal during viral infection is commonplace, but precise mechanisms and biological significance are not always clear^75^. Our observations of Golgi dispersal during COV infection is consistent with several previous reports^63–66^. One group identified multiple SARS-COV-2 proteins that are sufficient to trigger Golgi dispersal, including M^64^. By contrast, using immunostaining and a cargo sorting assay, we observed accumulation of M at the cis-Golgi, which correlated with a failure of diverse cargo proteins to traffic beyond this compartment. Our observations are consistent with accumulating evidence that Golgi dispersal during viral infection impedes protein trafficking in the secretory pathway*.”

2. Key figures 4-6 show convincing results but fragmentation of trans Golgi is hard to see. Also, increased cholesterol densities in GM130+ punctae are difficult to notice. One question: Why are the IFA images in color only in the merged panels, and not in the panels depicting single signals? It would help to see the single signals in color. Alos, scale bars should be added to the panels.

We agree that scale bars are very important, so they are placed in the bottom right-hand corner of all merged images in Figure 4, Figure 5, and Figure 6. The reason for using inverted black and white images for the single channels is to make them easier to interpret, as there is good evidence that readers can better discern differences in this format, rather than different colours, or a traditional light-coloured-image-on-dark-background format fluorescence microscopy image. We have now simplified the TNF-RUSH cargo sorting assay images in Figure 5 for ease of interpretation and have applied a Pearson’s Correlation Co-efficient test to convincingly demonstrate that SARS-CoV-2 M and the mApple-TNF-RUSH reporter accumulate together in the *cis*-Golgi following biotin addition.

We have also revised Figure 6 extensively to provide additional quantitative evidence for co-localization of CoV M and cholesterol at the *cis*-Golgi, as expressed through a Pearson’s Correlation Co-efficient test.

First, we obtained better representative micrographs shown in panel B, which clearly indicate a striking co-localization of the M-eGFP fusion protein with the mCherry-D4H fusion protein (**new Fig. 6B**) that is routinely used to visualize the trafficking of cholesterol at internal membranes, a kind gift provided by our colleague Dr. Fairn. We explored several methods for image quantitation and settled on a Pearson’s correlation coefficient (*r*) that demonstrates a significant co-localization of m-eGFP and mCherry-D4H (**new Fig. 6C**). Note that a value of 1 indicates a perfect positive linear relationship. Visual inspection of these images shows that even though immunostaining of the GM130 cis-Golgi marker was significantly less intense, this signal largely overlaps with both M-eGFP and mCherry-D4H (**new Fig. 6B**).

Importantly, we corroborated these findings using a different cholesterol probe, BODIPY 480/508-cholesterol (BCh2) that displays an excitation max of 480 nm and emission max of 508 nm. This green fluorescent cholesterol probe was loaded into cells expressing SARS-CoV-2 M fused to mCardinal and processed for confocal microscopy. We observed a striking co-localization of M and the BCh2 cholesterol probe (new **Fig. 6D**), which we confirmed using a Pearson’s correlation coefficient (new **Fig. 6E**). Together, and combined with our biochemical fractionation analysis in **Fig. 6A**, these approaches indicate aberrant cholesterol accumulation with SARS-CoV-2 M in detergent resistant membranes in the *cis*-Golgi.

**mCherry-D4H Refs:**

Maekawa M., Fairn G.D. Complementary probes reveal that phosphatidylserine is required for the proper transbilayer distribution of cholesterol. J. Cell Sci. 2015;128:1422–1433. doi: 10.1242/jcs.164715.

Maekawa M., Lee M., Wei K., Ridgway N.D., Fairn G.D. Staurosporines decrease ORMDL proteins and enhance sphingomyelin synthesis resulting in depletion of plasmalemmal phosphatidylserine. Sci. Rep. 2016;6:35762. doi: 10.1038/srep35762.

Cho K., van der Hoeven D., Zhou Y., Maekawa M., Ma X., Chen W., Fairn G.D., Hancock J.F. Inhibition of acid sphingomyelinase depletes cellular phosphatidylserine and mislocalizes K-Ras from the plasma membrane. Mol. Cell. Biol. 2016;36:363–374. doi: 10.1128/MCB.00719-15.

**BODIPY-Cholesterol (BCh2) Refs:**

Hölttä-Vuori, M.; Uronen, R.L.; Repakova, J.; Salonen, E.; Vattulainen, I.; Panula, P.; Li, Z.; Bittman, R.; Ikonen, E. BODIPY-cholesterol: A new tool to visualize sterol trafficking in living cells and organisms. *Traffic***2008**, *9*, 1839–1849.

Hölttä-Vuori, M.; Sezgin, E.; Eggeling, C.; Ikonen, E. Use of BODIPY-Cholesterol (TF-Chol) for Visualizing Lysosomal Cholesterol Accumulation. *Traffic* **2016**, *17*, 1054–1057.

Wüstner, D.; Lund, F.W.; Röhrl, C.; Stangl, H. Potential of BODIPY-cholesterol for analysis of cholesterol transport and diffusion in living cells. *Chem. Phys. Lipids* **2016**, *194*, 12–28.

Solanko, L.M.; Honigmann, A.; Midtiby, H.S.; Lund, F.W.; Brewer, J.R.; Dekaris, V.; Bittman, R.; Eggeling, C.; Wustner, D. Membrane orientation and lateral diffusion of BODIPY-cholesterol as a function of probe structure. *Biophys. J.* **2013**, *105*, 2082–2092.

3. While most of the submission is very clear, the discussion paragraph on lines 331-344 confuses this reviewer. This paragraph infers a relationship between broadly dispersed COPI, M accumulation in cis-Golgi, liquid ordered membranes in the cis-Golgi, TGN dispersal, and spike palmitoylation. There are many components here and this reviewer struggles with the alleged connections. This paragraph is potentially valuable and therefore it deserves additional clarifying text.

We agree.

We have revised and expanded this paragraph to improve clarity, as follows (lines 348-358, new text underlined):

“*In a recent study of global subcellular protein reorganization during HCOV-OC43 infection, all seven protein subunits of the COPI complex were mislocalized, which was confirmed by demonstrating that COPE and COPB2 redistribute from a perinuclear location to a broadly dispersed cytoplasmic pattern during infection^81^. Since COPI is responsible for intra-Golgi trafficking, and we have shown that M causes accumulation of protein cargo and cholesterol at the cis-Golgi, we speculate that M may increase the presence of liquid-ordered domains at the cis-Golgi. The formation of COPI vesicles occurs at liquid-disordered regions of the cis-Golgi, and cholesterol is largely excluded from these vesicles^82^. We speculate that recruitment of cholesterol by M could support virus assembly at the expense of COPI recruitment to this site, potentially impairing Golgi protein trafficking and triggering TGN dispersal. This speculation is supported by reports that M binds the Arf1 GTPase that recruits COPI to the cis-Golgi for vesicle formation^83^.*

4. One further comment related to #3; as authors speculate on mechanisms by which M proteins accumulate in cis-Golgi (linkage to ceramide-1-P was brought up) and impair cargo trafficking through Golgi, has this recent paper describing M-Arf1 interactions been considered (<https://doi.org/10.1038/s41467-025-61431-8> ) ?

We thank the reviewer for this helpful suggestion. Indeed, Arf1-mediated formation of COP-I vesicles is likely relevant to the mechanism of action of M. We think this is a potential avenue for future research, and we have added a section to the Discussion to address this paper, as follows (lines 354-358):

*“The formation of COPI vesicles occurs at liquid-disordered regions of the cis-Golgi, and cholesterol is largely excluded from these vesicles^82^. We speculate that recruitment of cholesterol by M could support virus assembly at the expense of COPI recruitment to this site, potentially impairing Golgi protein trafficking and triggering TGN dispersal. This speculation is supported by reports that M binds the Arf1 GTPase that recruits COPI to the cis-Golgi for vesicle formation^83^.”*

Minor concerns: none

**Reviewer #2:**

Caddell et al. present data supporting a model in which the Membrane (M) protein of SARS-CoV-2 (and other coronaviruses) disrupts the trans-Golgi network (TGN) such that secretory membrane trafficking is impaired. Consequently, the unfolded protein response (UPR) mediated by ATF6 is inhibited. The authors also show that this arm of the UPR as well as those mediated by PERK and IRE1 are triggered by Spike, but only the activation of ATF6 is inhibited my M, consistent with their model in that only ATF6 requires secretory transport for activation. The inhibition of ER-localized sensors by M extends to SREBP2 and STING, again consistent with the proposed mechanism, although sensing by these cellular proteins of coronavirus proteins or replication was not shown. Lastly, the authors note modulation of cholesterol at the cis-Golgi by M and propose that this is the likely mechanism by which M disrupts the TGN. Overall, the study is well-presented, and the conclusions follow the data. As noted below, some of the imaging data is not fully convincing.

Major issues:

1. Figure 4, which shows microscopically the localization of M with respect to cellular markers and the dispersal of the TGN by M, has no quantitation. At the least, co-localization data regarding M and the markers of the cis- and trans-Golgi should be generated. I wish I had a good suggestion for how to quantify TGN-dispersal, but perhaps the authors can think of a way to do it. One way might be to pick similar juxatanuclear areas in cells and determine the signal intensity (i.e., the signal density) of the TGN marker, since it appears to drop when M is expressed.

We have made conscious efforts to improve the presentation of microscopy data throughout the manuscript and have provided new quantitative data in Figure 5 (mApple-TNF-RUSH co-localization with M-EGFP upon biotin-mediated release of the cargo reporter from the ER) and Figure 6 (cholesterol probes and M), in the form of Pearson’s Correlation Co-efficient tests.

2. Figure 5, which uses the RUSH assay, is not convincing. Peripheral puncta appear present in the no-biotin condition, rendering the interpretation difficult. The choice of TNF as an indicator protein might not have been optimal. Wouldn't a protein like VSV-G, which would reach the plasma membrane when released from the ER, have been a better indicator?

As we stated on lines 247-254 in the Results (next text underlined), “*We observed that the reporter construct accumulated in the ER as expected, both in the presence of M or empty vector (EV) control, with punctate structures representing previously described ER exit sites (ERESs) and no signal overlap with the cis-Golgi compartment^68^ (****Fig 5B****). After 24 h of biotin treatment in EV transfected control cells, the reporter protein formed large puncta that partially co-localized with the cis-Golgi marker, displaying proper translocation and trafficking through the secretory pathway, as well as more distal puncta closer to the cell periphery (indicated by black arrows), indicative of vesicular trafficking toward the cell surface^67^.*

Reference 68 is a 2021 *Cell* paper from Jennifer Lippincott-Schwartz’s group that convincingly demonstrated that these puncta in the untreated (no biotin) control cells are ER exit sites (as shown in Supplemental figure below).

Ref 68:

Weigel AV, Chang CL, Shtengel G, Xu CS, Hoffman DP, Freeman M, Iyer N, Aaron J, Khuon S, Bogovic J, Qiu W, Hess HF, Lippincott-Schwartz J. ER-to-Golgi protein delivery through an interwoven, tubular network extending from ER. Cell. 2021 Apr 29;184(9):2412-2429.e16. doi: 10.1016/j.cell.2021.03.035. Epub 2021 Apr 13. PMID: 33852913.

Importantly, we have now used a Pearson’s Correlation Co-efficient test to quantify the co-localization of M and the mApple-TNF-RUSH reporter protein in the *cis*-Golgi. These proteins only co-localize following biotin addition.

We have revised Figure 5 to make it easier for the reader to interpret, removing the 4 h time point that provided little insight compared to the later 24 h time point.

3. Figure 6, which examines the effect of M on cholesterol in the cis-Golgi, is also not fully convincing or clear. In panel C, what are the four individual graphs representing? The control of rotating one channel is interesting, but why not obtain a Pearson's coefficient of correlation between the signals? Panel D lacks quantitation.

We have revised Figure 6 extensively to provide additional quantitative evidence for co-localization of CoV M and cholesterol at the *cis*-Golgi.

First, we obtained better representative micrographs shown in panel B, which clearly indicate a striking co-localization of the M-eGFP fusion protein with the mCherry-D4H fusion protein (**new Fig. 6B**) that is routinely used to visualize the trafficking of cholesterol at internal membranes, a kind gift provided by our colleague Dr. Fairn. We explored several methods for image quantitation and settled on a Pearson’s correlation coefficient (*r*) that demonstrates a significant co-localization of m-eGFP and mCherry-D4H (**new Fig. 6C**). Note that a value of 1 indicates a perfect positive linear relationship. Visual inspection of these images shows that even though immunostaining of the GM130 cis-Golgi marker was significantly less intense, this signal largely overlaps with both M-eGFP and mCherry-D4H (**new Fig. 6B**).

Importantly, we corroborated these findings using a different cholesterol probe, BODIPY 480/508-cholesterol (BCh2) that displays an excitation max of 480 nm and emission max of 508 nm. This green fluorescent cholesterol probe was loaded into cells expressing SARS-CoV-2 M fused to mCardinal and processed for confocal microscopy. We observed a striking co-localization of M and the BCh2 cholesterol probe (new **Fig. 6D**), which we confirmed using a Pearson’s Correlation Co-efficient test (new **Fig. 6E**). Together, and combined with our biochemical fractionation analysis in **Fig. 6A**, these approaches indicate aberrant cholesterol accumulation with SARS-CoV-2 M in detergent resistant membranes in the *cis*-Golgi.

**mCherry-D4H Refs:**

Maekawa M., Fairn G.D. Complementary probes reveal that phosphatidylserine is required for the proper transbilayer distribution of cholesterol. J. Cell Sci. 2015;128:1422–1433. doi: 10.1242/jcs.164715.

Maekawa M., Lee M., Wei K., Ridgway N.D., Fairn G.D. Staurosporines decrease ORMDL proteins and enhance sphingomyelin synthesis resulting in depletion of plasmalemmal phosphatidylserine. Sci. Rep. 2016;6:35762. doi: 10.1038/srep35762.

Cho K., van der Hoeven D., Zhou Y., Maekawa M., Ma X., Chen W., Fairn G.D., Hancock J.F. Inhibition of acid sphingomyelinase depletes cellular phosphatidylserine and mislocalizes K-Ras from the plasma membrane. Mol. Cell. Biol. 2016;36:363–374. doi: 10.1128/MCB.00719-15.

**BODIPY-Cholesterol (BCh2) Refs:**

Hölttä-Vuori, M.; Uronen, R.L.; Repakova, J.; Salonen, E.; Vattulainen, I.; Panula, P.; Li, Z.; Bittman, R.; Ikonen, E. BODIPY-cholesterol: A new tool to visualize sterol trafficking in living cells and organisms. *Traffic***2008**, *9*, 1839–1849.

Hölttä-Vuori, M.; Sezgin, E.; Eggeling, C.; Ikonen, E. Use of BODIPY-Cholesterol (TF-Chol) for Visualizing Lysosomal Cholesterol Accumulation. *Traffic* **2016**, *17*, 1054–1057.

Wüstner, D.; Lund, F.W.; Röhrl, C.; Stangl, H. Potential of BODIPY-cholesterol for analysis of cholesterol transport and diffusion in living cells. *Chem. Phys. Lipids* **2016**, *194*, 12–28.

Solanko, L.M.; Honigmann, A.; Midtiby, H.S.; Lund, F.W.; Brewer, J.R.; Dekaris, V.; Bittman, R.; Eggeling, C.; Wustner, D. Membrane orientation and lateral diffusion of BODIPY-cholesterol as a function of probe structure. *Biophys. J.* **2013**, *105*, 2082–2092.

4. The authors do not provide evidence that perturbations of cholesterol cause by M are causative with regard to TGN dispersal; both can be true but not causally related.

This is true.

We observe clear TGN dispersal in SARS-CoV-2 and HCoV-OC43 infected cells, and in SARS-CoV-2 M expressing cells, but the accumulation of cholesterol in the cis-Golgi is not necessarily causal for this phenotype. Proving this kind of causal relationship is extremely challenging, because it requires experimentally depleting cholesterol from the cis-Golgi compartment. We discuss mechanisms of COP-I vesicle formation that require cholesterol poor membrane substrates, and the role of COP-I vesicles in maintaining proper trafficking and structural organization of the Golgi. This is a fascinating focus for future research, which could involve approaches like drug-inducible degradation of M to observe the kinetics of TGN restoration and cholesterol redistribution (if such a thing could occur).

Minor Issues:

1.Figure 1E: by eye one would think that ORF10 is activating ATF6 and that M is inhibiting that, but those values are labelled as "ns" (not significant). Is that correct?

This is correct. We agree that by eye, it looks like ORF10 is significantly activating ATF6, but our statistical analysis does not support this conclusion. We applied an ANOVA statistical test for this large dataset, and the p-value for ORF10 data was 0.0777.

2.In supplemental Figure 2 it does not appear that infection with hCOV-229E is dispersing the TGN.

While the TGN is not completely disappearing in the hCoV-229E infected cells (below), the compartment is certainly altered in way that fits the term “dispersed” very well. Based on our experience with all three coronaviruses (SARS-CoV-2, hCoV-229E, hCoV-OC43) examined in our study, we think that the extent of TGN dispersal and outright disappearance likely correlates with the kinetics of infection. For example, SARS-CoV-2 has the fastest replication kinetics in the cell lines used in our study, and it also shows the most dramatic TGN dispersal phenotype.

3.Lines 262-3, "...we did not find that M could similarly alter the lipid composition of the ERGIC." What is the basis for this statement in the data presented?

We agree that this statement, referring to the biochemical isolation and characterization of detergent resistant membranes, could have been more clearly described. We were trying to describe how SARS-CoV-2 Spike was previously shown to cause the hallmark ERGIC marker ERGIC53 to accumulate in detergent-resistant membranes, and that our new data (below) shows that ERGIC53 accumulates normally in liquid disordered domains in M-expressing cells, just as Calnexin does. We have revised our description of this experiment for greater clarity, as follows (lines 270-281, new text underlined):

“…*COPI-mediated transport mechanisms are sensitive to perturbations in levels of sphingolipids and cholesterol in the Golgi^71^. We hypothesized that M inhibits anterograde secretion by altering cholesterol abundance in the cis-Golgi. To determine whether M is present in liquid-ordered domains, we isolated detergent resistant membranes (DRMs) from cells transfected with EV or M and conducted immunoblotting for cellular targets. M was found to be present in both the top (DRMs) and bottom (detergent soluble membranes (DSMs)) fractions (****Fig 6A****). Calnexin was selected as a marker for liquid disordered membranes and Caveolin was selected as a positive control marker for DRMs. Spike, which is heavily palmitoylated, was previously shown to increase the ordered lipid environment of the ERGIC causing accumulation of the ERGIC53 lectin in DRMs^72^. By contrast, M had no obvious effect on the lipid composition of the ERGIC, with ERGIC53 remaining associated with DSMs in M-expressing cells.*”

4. Line 900 has a grammatical error: "remained" could be replaced by "remainder" to correct this.

Fixed.

**Reviewer #3:**

The study examines effects of Coronavirus proteins on ER stress, golgi structure and secretory function.  Specifically, they look for effects on UPR activation, golgi dispersement and protein trafficking in the secretory pathway. They found that Spike protein triggered UPR whereas M protein inhibited the ATF6 pathway and blocked anterograde trafficking of UPR sensor proteins.  They observe cholesterol accumulation in the cis Golgi and dispersing of the trans Golgi suggesting the virus could be a way to interfere with host response to infection while maintaining ability to use cell to make nascent virions.    The study expands on other observations of corona virus proteins altering ER stress response and identifies M protein as a regulator of the ATF6 pathway or more generally altering the anterograde trafficking of proteins in the secretory pathway.  One of the concerns is the use of an enzymatic activity (luciferase) of a reporter protein to measure changes in proteostasis where the activity could be due to protein misfolding and not transcriptional changes.  Assessment of general cell viability would also be useful to rule out that decreases are not due to cytotoxicity.  Abbreviations should be clearly defined especially the use of “EV” which was interpreted to be extracellular vesicle prep from virally infected cells.  Overall, the findings appear novel and expand our understanding of corona virus M protein in altering cellular physiology.

Major issues:

What is EV?  Extracellular vesicles?  Not defined or referenced.  It says “transfected with EV” but then refers to “EV” cells elsewhere.   Also not clear what EV is from the methods/materials. Given this reviewer was delayed in reviewing this paper for variety of reasons, I will provide additional comments without fully understanding EV and assuming it is related to extracellular vesicles from infected cells.

We apologize for this unclear notation that was not properly defined. EV refers to “empty vector”, the matched empty plasmid vector used as a control in our transfection-based experiments throughout the manuscript. We now properly define this acronym upon first use in the Figure Legends.

In Figure 1, was there any independent verification that alterations of ATF6 pathway occurred using an endogenous marker (protein or RNA)?  Not necessarily needed for all proteins but for M effects?

We demonstrated specificity of the ESRE-luciferase assay for ATF6-N-mediated activation in Supplementary Figure 1, which showed that reporter assay activation in response to thapsigargin (Tg) treatment or SARS-CoV-2 Spike expression was dependent on the presence of the ATF6 gene; no luciferase was produced in the matched clonal ATF6 knockout cells that we generated.

The effects of M on ATF6 activation and processing was presented in Fig. 2E, while Fig. 2F shows that a plasmid-derived ATF6-N protein that doesn’t require trafficking in the secretory pathway for processing and activation is resistant to the effects of SARS-CoV-2 M protein.

In terms of the responses of endogenous proteins, ATF6-N is the primary stress-induced transcription factor for the ER chaperone BiP, and we show that endogenous levels of BiP protein are increased in response to Spike expression, which is reversed if M is co-transfected with Spike (Fig. 2C, at right).

Together, these experiments demonstrate the specificity of our ATF6-dependent functional assays in this manuscript.

Figure 1, could the decrease in luciferase be due to luciferase misfolding and not a reduction in the transcription of the ERSE reporter since protein folding and processing may be altered? For example, consider examining Luc mRNA expression by qPCR to show that it is also reduced for at least the strongest effect. 

The firefly luciferase reporter gene in the pESRE-luc plasmid is transactivated by ATF6-N and firefly luciferase accumulates in the cytoplasm of the transfected cell until it is released by lysis and processing for a Dual Luciferase Reporter Assay (Promega). The transfection control Renilla luciferase also accumulates in the cytoplasm of these cells, and it serves as the denominator in calculations of relative luciferase activity (ESRE-luc/Renilla-luc). The reviewer suggests that when M selectively inhibits accumulation of functional firefly luciferase in cells expressing SARS-CoV-2 M that this could be due to protein misfolding. But it is difficult to imagine how an ER/Golgi-localized M protein could interfere with firefly luciferase protein folding in the cytoplasm, and if it could, then why Renilla luciferase would not be similarly misfolded. Moreover, Fig 2B, at right, clearly shows that M alone does not cause ER stress or activation of any arm of the unfolded protein response.

The Tg responses in Figure 1F seem relatively low.  Also, could the low activity once again be due to M causing misfolding of the reporter?  Or high toxicity levels to the cells?  What is the Tg/Tm response alone?  And was the EV and M response alone ran in parallel?  These data are difficult to interpret without the controls. In Fig 1F, why are doses presented high to low (opposite to typical dose response and counterintuitive). 

In response to this critique, we have changed the order of values on the x-axis to move from low levels of Tg or Tm drug on the left to higher levels on the right (shown below). Hopefully, this is more intuitive for readers. As mentioned above, selective misfolding of a firefly luciferase reporter in the cytoplasm with no effect on Renilla luciferase is very unlikely. This is an extensively controlled experiment, with empty vector and mock-treated normalization controls.

Figure 2D, as the authors state in methods this is a semiquantitative assay and it is not clear what is being calculated in the right panel.  Also not clear is how many replicates were used (states n=3, is that 3 wells or 3 independent experiments?

This semiquantitative XBP1 mRNA splicing assay is a longstanding workhorse assay in this field that allows assessment of IRE1 activity by comparing levels of precursor (XBP1unspliced) to product (XBP1spliced). The graph was generated by capturing a digital image of the ethidium bromide stained DNA bands in the agarose gel and conducting densitometric analysis on these bands to yield numerical values. Thus, in the empty vector (EV) control lane, dividing the value of the single XBP1s band by the combined value of the XBP1u1 and XBP1u2 bands that result from cleavage with the PstI restriction endonuclease, yields a numerical ratio. This is set to 1. Applying this method to the data from the other lanes allows these values to be directly compared to the empty vector control. N=3 refers to three independent biological replicates, which is why there are three data points for each condition. We have now updated the Methods to provide more detailed information about how these values were obtained, as follows (lines 524-527, new text underlined):

“*Quantification of restriction digested PCR products were determined via densitometry using Image Lab 6.1 software (Bio-Rad). The ratio of XBP1s to XBP1u1 and XBP1u2 in all conditions was calculated by dividing XBP1s by XBP1u1+XBP1u2, these values are then normalized to the EV condition and plotted.”*

Figure 2F, the results (line 159) states the cells were ATF6 KO cells but figure legend states HEK293T cells.  Which one was used?  What is the reference for ATF6 KO cells?

We apologize for this mistake. The figure legend has now been modified to indicate that the cell line used in Fig. 2F is the clonal HEK293T-ATF6-KO cell line that we created, which is described in the Materials and Methods and validated in Supplementary Figure 1.

Figure 3.  Same concerns about protein folding of the different luciferases since activity is the endpoint measure and protein folding is being disrupted.

Fig 2B clearly shows that M alone does not cause ER stress or activation of any arm of the unfolded protein response. This directly addresses the critique about Fig. 3 and the speculation about possible effects of M on folding of any of the luciferase reporter enzymes, including the Gaussia luciferase reporter that traverses the secretory pathway (unlike the cytoplasmic firefly luciferase or Renilla luciferase reporters).

Quantitation of colocalization should be considered.  Pearson’s or Manders’ correlation plots should be straight forward with data presented.

We have revised Figure 6 extensively to provide additional quantitative evidence for co-localization of CoV M and cholesterol at the *cis*-Golgi.

First, we obtained better representative micrographs shown in panel B, which clearly indicate a striking co-localization of the M-eGFP fusion protein with the mCherry-D4H fusion protein (**new Fig. 6B**) that is routinely used to visualize the trafficking of cholesterol at internal membranes, a kind gift provided by our colleague Dr. Fairn. We explored several methods for image quantitation and settled on a Pearson’s correlation coefficient (*r*) that demonstrates a significant co-localization of m-eGFP and mCherry-D4H (**new Fig. 6C**). Note that a value of 1 indicates a perfect positive linear relationship. Visual inspection of these images shows that even though immunostaining of the GM130 cis-Golgi marker was significantly less intense, this signal largely overlaps with both M-eGFP and mCherry-D4H (**new Fig. 6B**).

Importantly, we corroborated these findings using a different cholesterol probe, BODIPY 480/508-cholesterol (BCh2) that displays an excitation max of 480 nm and emission max of 508 nm. This green fluorescent cholesterol probe was loaded into cells expressing SARS-CoV-2 M fused to mCardinal and processed for confocal microscopy. We observed a striking co-localization of M and the BCh2 cholesterol probe (new **Fig. 6D**), which we confirmed using a Pearson’s correlation coefficient (new **Fig. 6E**). Together, and combined with our biochemical fractionation analysis in **Fig. 6A**, these approaches indicate aberrant cholesterol accumulation with SARS-CoV-2 M in detergent resistant membranes in the *cis*-Golgi.

**mCherry-D4H Refs:**

Maekawa M., Fairn G.D. Complementary probes reveal that phosphatidylserine is required for the proper transbilayer distribution of cholesterol. J. Cell Sci. 2015;128:1422–1433. doi: 10.1242/jcs.164715.

Maekawa M., Lee M., Wei K., Ridgway N.D., Fairn G.D. Staurosporines decrease ORMDL proteins and enhance sphingomyelin synthesis resulting in depletion of plasmalemmal phosphatidylserine. Sci. Rep. 2016;6:35762. doi: 10.1038/srep35762.

Cho K., van der Hoeven D., Zhou Y., Maekawa M., Ma X., Chen W., Fairn G.D., Hancock J.F. Inhibition of acid sphingomyelinase depletes cellular phosphatidylserine and mislocalizes K-Ras from the plasma membrane. Mol. Cell. Biol. 2016;36:363–374. doi: 10.1128/MCB.00719-15.

**BODIPY-Cholesterol (BCh2) Refs:**

Hölttä-Vuori, M.; Uronen, R.L.; Repakova, J.; Salonen, E.; Vattulainen, I.; Panula, P.; Li, Z.; Bittman, R.; Ikonen, E. BODIPY-cholesterol: A new tool to visualize sterol trafficking in living cells and organisms. *Traffic***2008**, *9*, 1839–1849.

Hölttä-Vuori, M.; Sezgin, E.; Eggeling, C.; Ikonen, E. Use of BODIPY-Cholesterol (TF-Chol) for Visualizing Lysosomal Cholesterol Accumulation. *Traffic* **2016**, *17*, 1054–1057.

Wüstner, D.; Lund, F.W.; Röhrl, C.; Stangl, H. Potential of BODIPY-cholesterol for analysis of cholesterol transport and diffusion in living cells. *Chem. Phys. Lipids* **2016**, *194*, 12–28.

Solanko, L.M.; Honigmann, A.; Midtiby, H.S.; Lund, F.W.; Brewer, J.R.; Dekaris, V.; Bittman, R.; Eggeling, C.; Wustner, D. Membrane orientation and lateral diffusion of BODIPY-cholesterol as a function of probe structure. *Biophys. J.* **2013**, *105*, 2082–2092.

For Figure 6, it is not clear whether the location of sensor or the location of cholesterol is being affected by M protein.  Is there at difference from M-eGFP and MeGFP (to clarify there isn’t a M-EGFP fusion).  Both are mentioned in the results section.

We apologize for these inconsistencies in naming the M-EGFP construct in the manuscript; we have now harmonized this naming for consistency throughout the manuscript.

We studied the subcellular localization of untagged SARS-CoV-2 M by immunostaining, as well as fusions with fluorescent proteins where EGFP or mCardinal are fused to the cytosolic carboxy-terminus of M. These proteins all accumulate in the cis-Golgi in transfected cells as indicated in our images. We have now corroborated these findings using a second probe, BODIPY 480/508-cholesterol (BCh2) (new **Figs. 6D, 6E**) that accumulates in the cis-Golgi only in the presence of M. While it is not possible to visualize endogenous intracellular cholesterol directly in this compartment, the perfect concordance of the chemical BCh2 probe with the plasmid-derived D4H-mCherry probe provides confidence for cholesterol accumulation at this intracellular compartment.

Minor issues:

Check document for spelling, grammar, font, etc.

Fixed.

We have extensively revised the manuscript to fix any errors in spelling, grammar, etc.
